# Spironolactone-Loaded LeciPlexes as Potential Topical Delivery Systems for Female Acne: In Vitro Appraisal and Ex Vivo Skin Permeability Studies

**DOI:** 10.3390/pharmaceutics12010025

**Published:** 2019-12-25

**Authors:** Ayman Salama, Mohamed Badran, Mohammed Elmowafy, Ghareb M. Soliman

**Affiliations:** 1 Department of Pharmaceutics, Faculty of Pharmacy, University of Tabuk, Tabuk 71491, Saudi Arabia; 2 Department of Pharmaceutics and Industrial Pharmacy, Faculty of Pharmacy (Boys), Al-Azhar University, Nasr City, Cairo 11751, Egypt; mbadran75@gmail.com (M.B.); morere_om@outlook.com (M.E.); 3 Department of Pharmaceutics, Faculty of Pharmacy, Assiut University, Assiut 71526, Egypt

**Keywords:** LeciPlex, spironolactone, female acne, topical delivery

## Abstract

Spironolactone (SP), an aldosterone antagonist with anti-androgen properties, has shown promising results in the treatment of female acne. However, its systemic side effects limit its clinical benefits. This study aimed to prepare and evaluate LeciPlexes for SP topical delivery. LeciPlexes were prepared by a one-step procedure and characterized using various techniques. Optimum LeciPlex preparation was incorporated into 1% methylcellulose gel and SP permeability was tested ex vivo in Sprague-Dawley rat skin. The maximum drug encapsulation efficiency obtained was 93.6 ± 6.9% and was dependent on the drug/phospholipid and surfactant/phospholipid ratios. A zeta potential of +49.3 ± 3.5 to +57.7 ± 3.3 mV and a size of 108 ± 25.3 to 668.5 ± 120.3 nm were observed for the LeciPlexes. FT-IR and DSC studies confirmed the incorporation of SP into the LeciPlexes through hydrophobic and hydrogen bonding interactions. SP release from the LeciPlex formulations was significantly slower than from the drug suspension. Cumulative SP permeated through rat skin from LeciPlex gel was about 2-fold higher than SP control gel. Cumulative SP deposited in the stratum corneum and other skin layers from the LeciPlex gel was about 1.8- and 2.6-fold higher than SP control gel, respectively. This new SP LeciPlex formulation is a promising carrier for the treatment of female acne.

## 1. Introduction

Acne vulgaris is a highly prevalent chronic inflammatory condition of the skin affecting more than 80% of teenagers [1]. Acne peak incidence occurs in 14–17-year-old girls and 16–19-year-old boys [2]. About 15–20% of affected patients have moderate-to-severe acne [3]. Acne is classified by The Global Burden of Disease Study as the eighth most prevalent disease worldwide and the third most prevalent dermatological condition affecting about 10% of the global population [4,5]. It can persist beyond the age of 25 years, especially in women [2,6]. Perkins et al. reported that 45% of women aged 21–30, 26% of those aged 31–40, and 12% of those aged 41–50 had clinical acne [7]. The pathoetiology of acne is associated with factors that cause changes in the natural skin barrier functions and microorganisms, leading to hyperseborrhea, changed keratinization of the pilosebaceous duct, loss of the skin microbial variety, and inflammation [8]. The main hormones implicated in the etiology of acne include androgens, insulin, and insulin-like growth factor-1 [1,9]. Increased sebum productions stimulates follicular hyperkeratinization, leading to microcomedo [10].

Although acne is not a life-threatening disease, its effect on the patient’s quality of life and general well-being are undoubtful. Acne has a negative impact on the patient’s quality of life, self-esteem, and mood, leading to increased risk of anxiety, depression, frustration, dissatisfaction with appearance, and suicidal thoughts [6,11]. This might lead to poor body image and limited social activities [6]. Female patients were found to have worse scores in the Dermatology Quality of Life Index and acne quality of life self-assessment score compared with male patients [2,3,12].

It is believed that hormones have a great influence on female acne, as evidenced by appearance of acne at menarche, increased sebum excretion, and premenstrual bursts, as well as hirsutism and ovarian cysts [13]. Androgen is essential for the development of acne. Therefore, decreasing androgen activity has been proposed as a treatment modality for female acne. Spironolactone (SP) is an aldosterone antagonist with anti-androgen properties [14]. It has been used as a potassium sparing diuretic since 1957. SP’s mechanism of action includes blocking 5α-reductase activity through increasing testosterone clearance as a result of enhanced liver hydroxylase activity [15]. SP was also shown to competitively inhibit androgens, mainly dihydrotestosterone and testosterone, through binding to androgen receptors of the skin, which decreases androgen-stimulated sebocyte proliferation. This results in reduced sebum production and improved acne symptoms [16,17]. Depending on the clinical situation, the dose of SP for the treatment of female acne ranges from 25 to 200 mg/day [15]. Although several clinical studies have confirmed the effectiveness of oral SP in the treatment of female acne, systemic side effects might restrict its clinical application. The most common side effects include diuretic effects, menstrual irregularity, breast tenderness and enlargement, dizziness, headaches, nausea, and vomiting [15,18,19,20].

Topical SP delivery is an appealing approach to circumvent these side effects, improve drug efficacy, and enhance patient compliance. Local drug application guarantees the presence of high drug concentrations at the disease site, which leads to reduced systemic side effects, improved patient compliance, reduced cost of therapy, and overall better therapeutic outcomes [21,22]. In a randomized double-blind clinical trial, Afzali et al. compared the efficacy of 5% SP gel to placebo in the treatment of mild-to-moderate acne [23]. Treatment outcome was evaluated by total acne lesions counting (TLC) and acne severity index (ASI). Patients taking SP gel had significantly lower TLC compared with the placebo group. However, the difference between ASI in the two groups was not significant.

Liposomes are phospholipid-based vesicular systems that have been extensively explored for skin delivery of various drugs [24,25]. Liposomes are able to encapsulate a wide range of drugs, including hydrophilic and hydrophobic ones, and enhance their delivery through the skin. LeciPlex is a fairly new vesicular nanodelivery system based on the combination of negatively charged phospholipids and cationic surfactants [26]. LeciPlexes are prepared by a simple one-step procedure and have a positive surface charge, which facilitates their intimate interaction with the negatively charged skin surface, leading to enhanced drug penetration into the deep skin layers [27]. Cationic surfactants such as cetyltrimethylammonium bromide (CTAB) used in the preparation of LeciPlex systems were reported to be cytotoxic to human skin HaCaT keratinocyte cells at a concentration as low as 10 µM [28]. However, previous studies showed that the association between CTAB and phospholipids in LeciPlex systems greatly limits their cytotoxic effects [26,29]. Moreover, the toxic effect of CTAB was reported to be due to free CTAB in solution, which is usually removed by centrifugation during LeciPlex preparation [28]. LeciPlex preparations were shown to enhance the bioavailability of various drugs. For instance, LeciPlexes improved the ocular bioavailability and transcorneal permeability of carvedilol [30]. They also improved the anti-inflammatory and antitumor activity of quercetin compared to drug suspension upon oral administration [31]. So far, there are no published reports on using LeciPlexes to enhance the anti-acne effects of SP.

There are only two published reports on the encapsulation of SP into lipid-based nanocarriers (e.g., solid lipid nanoparticles (SLNs) and nanosctructured lipid carriers (NLCs)) as a potential topical treatment of acne [32,33]. In a randomized, double-blind, prospective trial SP-NLCs and SP-alcoholic gel were able to significantly reduce the mean number of total acne lesions and non-inflammatory lesions [33].

The aim of this project was to prepare SP-loaded LeciPlexes as a potential treatment of female acne and test their ability to enhance the skin delivery of the drug. The LeciPlexes were prepared by a simple one-step procedure and evaluated using various techniques. The ex vivo skin permeability was evaluated using Sprague-Dawley rat skin. To the best of our knowledge, this is the first attempt to enhance the topical anti-acne effects of spironolactone through encapsulation into LeciPlex-based delivery systems.

## 2. Materials and Methods

### 2.1. Materials

Spironolactone was obtained from Shaanxi Pioneer Biotch Co., Xi’an, China. Soybean Lecithin (Phospholipon^®^ 90G, PL-90G) was a gift from Lipoid AG, Steinhausen, Switzerland. Diethylene glycol monoethyl ether (Transcutol^®^ P) was obtained from Gattefossé, Saint-Priest, France. Cetyltrimethylammonium bromide (CTAB) was purchased from Morgan Company for Chemicals (Cairo, Egypt). Methylcellulose was obtained from Sigma Aldrich Co., St. Louis, MO, USA.

### 2.2. Preparation of Drug-Loaded LeciPlexes

LeciPlexes were prepared by a one-step fabrication process using equimolar amounts of PL-90G and CTAB according to previously published procedures [26,27]. Briefly, the drug (10 mg) and PL-90G (0.182 g, 24 mM) were dissolved in Transcutol^®^ P (0.5 mL) by heating the mixture briefly to 70 °C in a water bath. CTAB (0.09 g, 24 mM) solution in water (9.5 mL, 70 °C) was added to the drug/PL-90G mixture under magnetic stirring to form SP-loaded LeciPlexes. The mixture was stirred continuously until a uniform dispersion was obtained.

Conventional liposomes were prepared and used as a control. PL-90G (24 mM) and SP (10 mg) were dissolved in Transcutol^®^ P (0.5 mL, 70 °C). Distilled water (9.5 mL maintained at 70 °C) was added to the drug/PL-90G mixture at once under vigorous stirring. The stirring was continued until a uniform dispersion was obtained [26].

### 2.3. Effect of Drug/Lipid Ratio on LeciPlex Properties

SP-loaded LeciPlexes were prepared as outlined above using various drug amounts (10, 20, 30, 40, and 50 mg) at CTAB/PL-90G molar ratio of 1:1, and their properties were evaluated.

### 2.4. Effect of CTAB/PL-90G Molar Ratio on LeciPlex Properties

SP-loaded LeciPlexes were prepared as outlined above using 10 mg SP and different CTAB/PL-90G molar ratios (0.25:1, 0.5:1, 1:1, 1.5:1, 2:1, 3:1). Various LeciPlex properties were evaluated.

### 2.5. Evaluation of SP-Loaded LeciPlexes 

#### 2.5.1. Determination of Drug Entrapment Efficiency 

NaCl (100 mg) was added to 1 mL of LeciPlex dispersion and centrifuged at 14,000 rpm for 30 min to separate the nanoparticles. NaCl was added to facilitate LeciPlex separation. The supernatant was separated and analyzed for unencapsulated drug spectrophotometrically at 238 nm and using a calibration curve (*R*^2^: 0.9998; limit of detection: 0.07 µg/mL; limit of quantification: 0.2 µg/mL). The drug encapsulation efficiency was calculated from the following equation [34]:

(1) $$\mathrm{Encapsulation}\;\mathrm{efficiency}\;\left(\mathrm{weight}\;\%\right)=\frac{T-C}{T}\;\times 100$$

where *T* is the total amount of SP used in LeciPlex preparation and *C* is the amount of SP in the supernatant.

#### 2.5.2. HPLC Assay of SP

Determination of SP was performed according to a previously reported method with minor modifications [35]. The HPLC system used was Waters^TM^ 600 controller equipped with a wavelength detector (Waters^TM^ 2487 Dual λ Absorbance detector, Milford, MA, USA), a pump (Waters^TM^ 1252 Binary pump, USA), and an automated sampling system (Waters^TM^ 717 Plus Autosampler, USA). The mobile phase (acetonitrile/water, 70:30, *v*/*v*) was flowed over a reversed-phase C18 column (µ-Bondapak^TM^, 4.6 × 150 mm, 5 µm particle size) at a rate of 1 mL/min at room temperature. Aliquot (20 μL) of the samples was injected and the drug was quantified by its UV absorbance at 238 nm and using a calibration curve (*R*^2^: 0.998; limit of detection: 0.14 µg/mL; limit of quantification: 0.43 µg/mL). The peak areas were integrated automatically by a computer using a software program “Empower (Waters^TM^)” software (Version 3, Empower software solutions Inc., Orlando, FL, USA, 2017).

#### 2.5.3. Determination of Particle Size and Zeta Potential

LeciPlex particle size and zeta potential were measured on a Malvern Nano-ZetaSizer (Nano-ZS, Malvern Instruments, Worcestershire, UK). The instrument had a He–Ne laser operating at 633 nm and an avalanche photodiode detector. Samples of the LeciPlex preparations were diluted 100-fold by distilled water and measured in triplicate at room temperature. A cumulant analysis was used to obtain the hydrodynamic diameter of the LeciPlexes. The zeta potential was calculated from the Smoluchowski equation [36].

#### 2.5.4. FT-IR Studies

Compatibility between SP and various LeciPlex components was investigated by Fourier transform infrared (FT-IR) in the transmission mode. FT-IR analysis was performed between 4400 and 400 cm^−1^ at a resolution of 4 cm^−1^ on a Nicolet iS10 FT-IR Spectrometer (Thermo Electron Scientific Corporation, USA). Lyophilized LeciPlex formulation (F1) was compared with samples of SP alone, PL-G90 alone, CTAB alone, and SP/PL-G90/CTAB physical mixture. The physical mixture had the same composition as LeciPlex formulation F1 and was used to make sure that any observed changes are due to drug incorporation into LeciPlexes.

#### 2.5.5. Differential Scanning Calorimetry (DSC) Studies

DSC studies were performed to further confirm the compatibility of SP with different LeciPlex components. Samples of freeze-dried LeciPlex F1, SP, PL-90G, CTAB, and SP/PL-90G/CTAB physical mixture were analyzed using a DSC instrument (DSC3, Mettler Toledo, Im Langacher, Switzerland). The physical mixture had the same composition as LeciPlex formulation F1. Accurately weighed 5 mg samples were placed in aluminum pans, crimped, and analyzed between 30 and 250 °C at a scan rate of 10 °C/min under constant nitrogen flow. An empty aluminum pan was used as a reference.

#### 2.5.6. TEM Measurements 

The morphology of SP-loaded LeciPlex F1 was observed by transmission electron microscopy (TEM; JEOL, JEM-1010S, Tokyo, Japan). LeciPlex F1 was diluted with distilled water and placed on a carbon-coated copper grid to form a thin liquid film. Excess of sample was then removed by a filter paper. The film on the grid was allowed to dry at room temperature, and then observed by TEM and photographed.

### 2.6. Formulation of LeciPlex Gel

Plain methylcellulose (MC) gel (1%, *w*/*v*) was prepared by gently dispersing the required amount of MC in 100 mL of boiling deionized water, followed by magnetic stirring at a high speed. Stirring was continued until a thin, lump-free hazy dispersion was obtained. The gel was left overnight in the refrigerator. To prepare SP LeciPlex-loaded gel, a given weight of the LeciPlex F1 pellet obtained after centrifugation of the LeciPlex dispersion was mixed with a given weight of MC plain gel (1%, *w*/*v*). Control SP gel was prepared by adding a given weight of SP to 100 mL of boiling deionized water, followed by the addition of the required MC weight, and the rest of the process was the same as the plain gel. SP concentration in all of the preparations was 1%, *w*/*v*.

### 2.7. In Vitro Drug Release Studies

In vitro SP release from LeciPlex F1 and LeciPlex F1 gel formulations was evaluated by a screw capped Spectra/Por^®^ dialyzing tube (Float-A-Lyzer^®^ G2 with Biotech Cellulose Ester Membrane, MWCO 8000–10,000 Da, Sigma Aldrich, St. Louis, MO, USA), tightly closed at both ends. The receptor medium was 200 mL of pH 7.4 phosphate-buffered saline. Formulations under investigation were placed in the dialyzing tubes, then closed by the caps. Tubes were immersed in the receptor media placed in closed beakers, adjusted at 37 ± 2 °C, and stirred at 100 rpm using magnetic stirring hot plates (Thermolyne Corporation, Waltham, MA, USA). At predefined time intervals, aliquots of 2 mL were withdrawn from the receptor media and analyzed spectrophotometrically at 238 nm. Equal volumes of fresh buffer were added to the receptor media immediately after sample withdrawal to maintain constant receptor volume. All of the experiments were done in triplicate. SP suspension (1 mg/mL) in 0.5%, *w*/*v* carboxymethylcellulose was similarly treated and used as a control.

### 2.8. Kinetic Treatment of the Release Data

Different drug release kinetic models were employed to analyze SP release data from the studied formulations to get insights into the mechanism of drug release. The utilized models were zero-order, first-order, Higuchi model, Baker–Lonsdale model, Hixson–Crowell cube root equation, and Korsmeyer–Peppas equation [37,38,39].

### 2.9. Ex Vivo Rat Skin Penetration Study

#### 2.9.1. Preparation of Rat Skin

Ex vivo permeability studies of SP-loaded LeciPlex were carried out using excised rat abdominal skin obtained from Sprague-Dawley male rats weighing 250–300 g. The hair was removed by an electric clipper, followed by removal of the subcutaneous fat. Skin was then cut into pieces and wrapped into an aluminum foil, followed by storage at −20 °C for future use. Skin pieces having any surface irregularities such as holes or crevices were excluded. The experiments of acquiring and processing animal skin were approved by the Institutional Animal Ethical Committee (approval number S4-19, 1-9-2019), which adheres to the Guide for the Care and Use of Laboratory Animals, 8th Edition, National Academies Press, Washington, DC, USA.

#### 2.9.2. Ex Vivo Permeation Experiments

Vertical Franz diffusion cells with a diffusion area of 1.76 cm^2^ were used for this study according to a previously published study [40]. Skin samples were thawed for 1 h immediately before the experiment and then hydrated using PBS for another hour. The receptor compartment contained 12.5 mL of pH 7.4 PBS. The buffer was degassed using a water bath sonicator for 30 min just before the experiment. Skin was mounted with stratum corneum side up. Care was taken to avoid air bubbles between the skin and diffusion medium during mounting. Skin wrinkles were avoided by uniformly stretching the skin specimen. Skin surface was maintained at 32 °C by circulating water at 37 °C through the outer jackets of the Franz diffusion chambers. SP-loaded LeciPlex F1 gel and SP gel (100 mg corresponding to 1 mg SP) were applied over the skin in the donor compartment in an occlusive manner. The dose was uniformly distributed on the skin surface with the aid of an inoculation loop. At different time intervals (0.5, 1, 2, 4, 6, 8, 24, 30, and 48 h), 1 mL samples were withdrawn from the receptor compartment and replaced by an equal volume of freshly prepared receptor medium. At the end of the study, the skin samples were carefully rinsed to discard the residue of preparations (0.5 mL water, 4 times). The skin samples were fixed onto cork plates and stretched using small pins. The stratum corneum was then removed by tape-stripping (3 M Transpore^TM^ tape, St. Paul, MN, USA). Tapes with a surface area of approximately 4.0 cm^2^ were applied on the stratum corneum of the skin. The tape was firmly pressed on the skin surface and pulled off immediately using a force of approximately 2 kg. Each skin sample was stripped with 10 tapes to confirm the removal of the stratum corneum [27]. The first tape was discarded to avoid any contamination from the applied formulation. The stripped skin was cut into small pieces, and the tapes and stripped skin pieces were placed separately in a solvent mixture of PBS pH 7.4/acetonitrile (1:2, *v*/*v*) overnight, followed by 5 min vortexing and 5 min sonication for complete extraction of SP. All of the samples were centrifuged at 25,000 rpm for 20 min. The supernatant was filtered through a 0.22 µm membrane filter. The amount of SP in extracts of the tapes and stripped skin, as well as the receptor fluids, were assayed by HPLC as described above. The validity of the extraction method was evaluated. More than 90% of SP was recovered from the spiked samples of the tapes and stripped skin. Cumulative SP amount permeated through skin (µg·cm^–2^) was calculated and plotted against time. The flux (*Js*, µg·cm^−2^·h^−1^) was taken as the slope of the linear regression line [41]. The apparent permeability coefficient (*P*_app_, cm·h^−1^) was calculated from Equation (2).

(2) $$P_{\mathrm{app}}=\;\frac{J_{S}}{C_{0}}$$

where *C*_0_ is the initial SP concentration in the donor compartment.

### 2.10. Statistical Analysis 

All experiments were performed in triplicate and the results are shown as mean ± standard deviation (SD). The data were analyzed using Graph-Pad Prism version 5 software (GraphPad Software, Inc., USA). One-way analysis of variance (ANOVA) with Newman–Keuls post-hoc test was used to evaluate the difference between means. Statistical significance was set at *p* < 0.05.

## 3. Results and Discussion 

### 3.1. Preparation of SP-Loaded LeciPlexes

#### 3.1.1. Effect of SP Concentration on LeciPlex Properties

The composition of various SP-loaded LeciPlex preparations is shown in Table 1. The LeciPlexes were prepared by a one-step nanoprecipitation method using the biocompatible solvent Transcutol^®^ P. This method allows incorporation of high drug concentrations and avoids the use of toxic organic solvents, such as acetone, dichloromethane, and chloroform [26]. Table 2 shows different LeciPlex properties as a function of their composition. In formulations F1–F5, the concentrations of PL-90G and CTAB were kept constant, both at 24 mM, while the amount of SP was varied between 10 and 50 mg. The drug encapsulation efficiency increased from 88.74 ± 3.4% to 93.6 ± 6.9% with the increase of SP from 10 to 30 mg (non-significant change, *p* > 0.05), after which it started to decrease. Thus, increasing the drug amount from 30 to 50 mg resulted in a significant decrease (*p* < 0.05) in the encapsulation efficiency from 93.6 ± 6.9% to 79.5 ± 7.6%. This indicates that maximum drug encapsulation was achieved at SP amount of 30 mg, after which excess drug was precipitated, leading to decreased encapsulation efficiency. Other drug-loaded LeciPlex systems showed drug encapsulation efficiency of ≥90% [30,31,42]. The increase in drug encapsulation was accompanied with an increase in particle size from 337 ± 11.06 to 591 ± 13.7 nm, after which the size decreased with further increase in drug concentration. The polydispersity index for all the preparations was between 0.3 and 0.9. Based on these results, formulation F1 that had the smallest particle size (337 ± 11.1 nm), smallest polydispersity index (0.3), and reasonably high drug encapsulation efficiency (88.74 ± 3.4%) was selected to study the effect of CTAB/PL-90G ratio on the LeciPlex properties (Table 1, F6–F10).

#### 3.1.2. Effect of CTAB/PL-90G Ratio on LeciPlex Properties

The CTAB/PL-90G molar ratio was varied from 0.25:1 to 3:1 and the LeciPlex properties were evaluated (Table 2). The drug encapsulation efficiency was generally increased with the increase in the CTAB/PL-90G ratio up to a ratio of 1.5:1, after which it started to decrease. For instance, there was a significant increase (*p* < 0.05) in the drug encapsulation efficiency from 85.3 ± 1.9% to 92.8 ± 5.7% with the increase of CTAB/PL-90G ratio from 0.25:1 to 1.5:1. This might be attributed to the ability of CTAB to enhance the drug incorporation into the phospholipid bilayer. Similar results were observed for the encapsulation of griseofulvin into deformable vesicles containing various surfactants as edge activators [43]. The drug encapsulation efficiency significantly decreased (*p* < 0.05) at CTAB/PL-90G ratios greater than 1.5:1, most probably due to lecithin solubilization by CTAB, leading to drug leakage from the LeciPlex [26]. The increase in CTAB/PL-90G molar ratio was accompanied by a significant decrease in the particle size (*p* < 0.05). A similar trend was previously observed for CTAB/lecithin nanoparticles [29].

The zeta potential of all the tested formulations was in the range of +49.3 ± 3.5 to +57.7 ± 3.3 mV, which confirms the colloidal stability of the nanoparticles. Zeta potential of ±30 mV or more is essential for nanoparticle colloidal stability through electrostatic repulsion [44]. The zeta potential of the LeciPlex formulations was almost constant for all of the formulations and did not change with the increase in CTAB content of the formulations. This is in agreement with previous reports, which showed that the zeta potential of lecithin-based nanoparticles did not increase at CTAB concentration larger than 7.5 mM [29]. This might indicate that the LeciPlexes have certain capacity for CTAB, after which the excess surfactant is not incorporated into them. In our case, most formulations had a CTAB concentration larger than 6 mM, which might explain the constant zeta potential (Table 1).

### 3.2. FT-IR Studies

**Figure 1:**
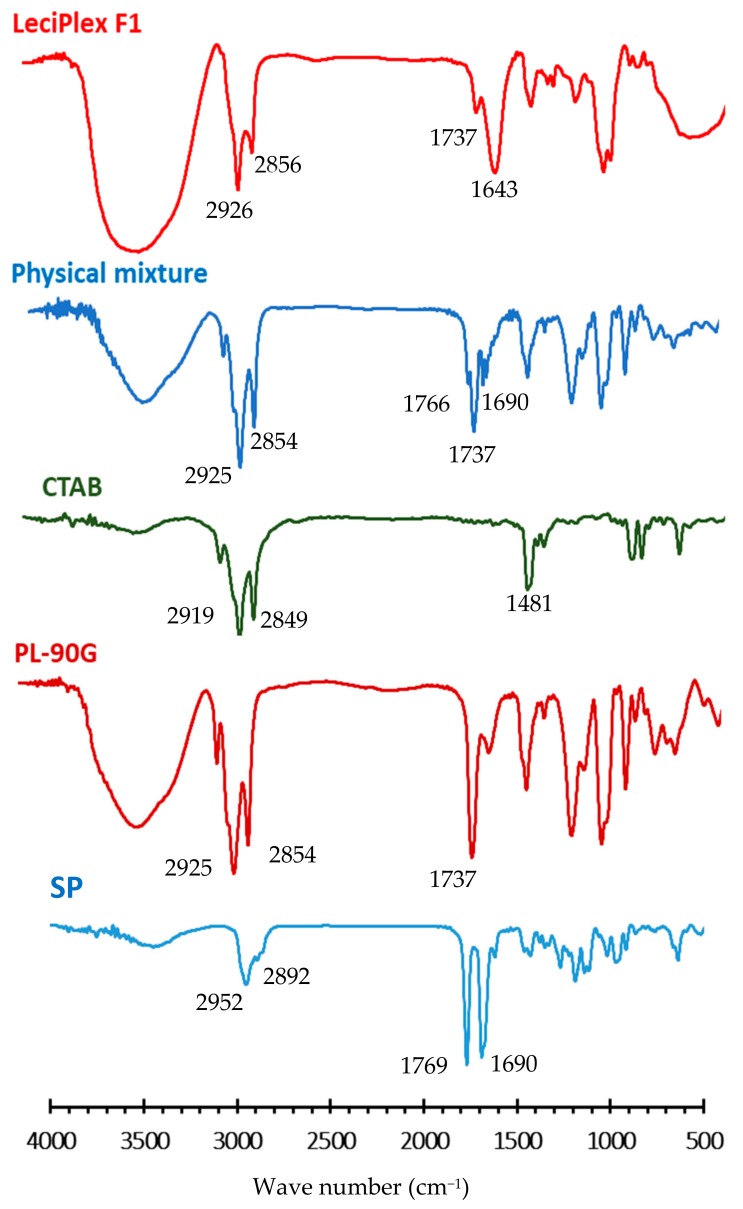
FT-IR spectra of spironolactone (SP), Phospholipon^®^ 90G (PL-90G), cetyltrimethylammonium bromide (CTAB), their physical mixture, and LeciPlex formulation F1.

SP FT-IR spectrum (Figure 1) shows the following characteristic absorption bands: a C–H stretching vibration band centered at 2952 cm^−1^, a band for stretching vibration of carbonyl group of lactone at 1769 cm^−1^, and a stretching vibration band for thioacetyl carbonyl group at 1690 cm^−1^ [45]. The spectrum of PL-90G (Figure 1) shows characteristic C–H stretching bands at 2925 and 2854 cm^−1^, a stretching band of ester carbonyl group at 1737 cm^−1^, and an ester C–O stretching band at 1240 cm^−1^ [34]. CTAB spectrum (Figure 1) shows C–H stretching vibrations at 2919 and 2849 cm^−1^ for methyl and methylene groups, respectively. The bands in the region of 1400–1500 cm^−1^ are due to C−H bending vibration [46]. The spectrum of SP/CTAB/PL-90G physical mixture (Figure 1) shows peaks at almost the same wave numbers as those of its components. In contrast, the spectrum of LeciPlexes F1 (Figure 1) shows a shift of the bands for the stretching vibration of SP lactone carbonyl group and thioacetyl carbonyl group to 1737 and 1643 cm^−1^, respectively. This might be taken as evidence of hydrophobic interactions and intermolecular hydrogen bonding between SP and LeciPlex components [32]. These interactions are essential for SP loading into the LeciPlex bilayer structure and for controlling its release.

### 3.3. DSC Studies 

**Figure 2:**
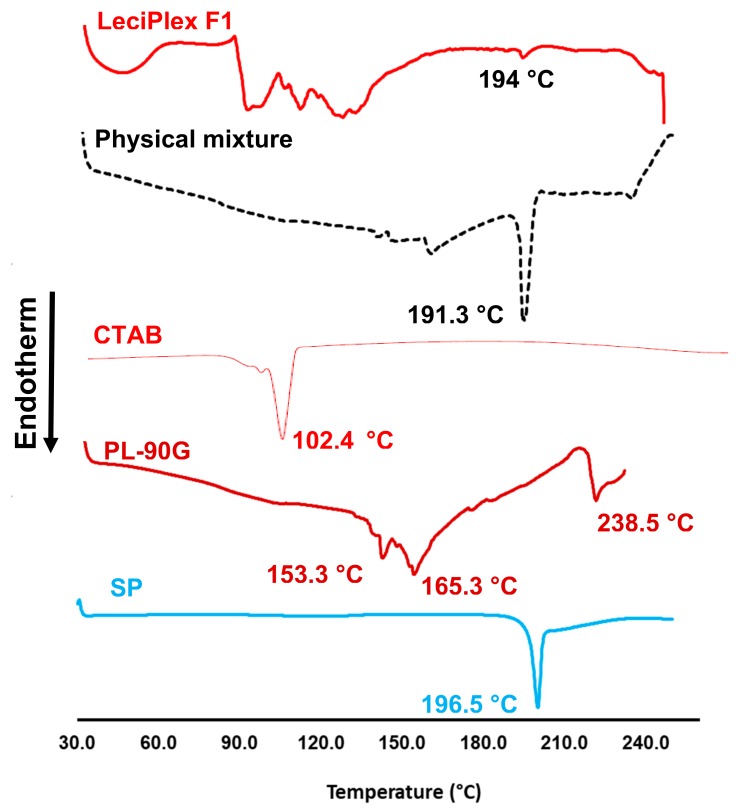
DSC thermograms of spironolactone (SP), Phospholipon^®^ 90G (PL-90G), CTAB, their physical mixture, and LeciPlex formulation F1.

To further confirm the interaction between SP and various LeciPlex components, DSC thermograms were recorded for SP, PL-90G, CTAB, their physical mixture and LeciPlex F1 (Figure 2). The SP thermogram shows a single sharp endotherm with an onset at 196.5 °C, corresponding to the drug melting point [47]. The thermogram of PL-90G shows three some-what broad peaks at 153.3, 165.3 and 238.5 °C, consistent with the thermal behavior of amorphous substances. The CTAB thermogram shows a sharp endotherm in the tested temperature range at 102.4 °C due to moisture loss [48]. The physical mixture shows the same peaks of SP and PL-90G, albeit the peaks were shifted to lower temperatures. However, the intensity of SP melting peak was not changed. This shift in peak position might be attributed to PL-90G melting before the drug, leading to partial or complete drug dissolution in the molten lipid, which in turn resulted in lower drug melting point [49]. The thermogram of LeciPlex F1 shows a series of broad peaks in the range of 94–133 °C, ascribed probably to CTAB and PL-90G thermal events. In addition, this thermogram shows a very tiny endothermic peak with an onset at 194 °C, which is probably attributed to the melting of SP. Similar to the thermogram of the physical mixture, there was a shift in the melting endotherm of SP. However, there was also a great reduction in the intensity of SP melting endotherm. The shift in position and decrease in intensity of SP peak might be taken as an evidence of hydrogen bonding and hydrophobic interactions between the drug and various LeciPlex components, which corroborates the results obtained from FT-IR studies. These interactions are essential to facilitate drug loading in the nanocarrier and also to sustain its release once it is administered in the human body.

### 3.4. TEM Measurements 

**Figure 3:**
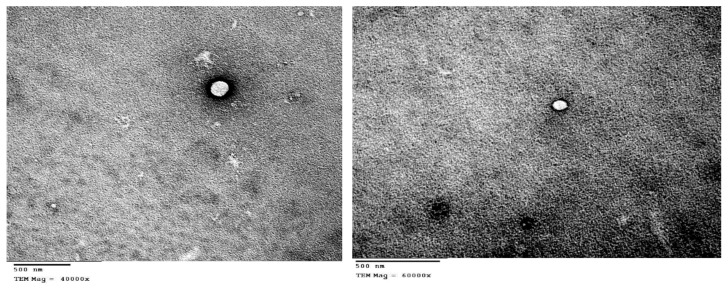
TEM photomicrograph of LeciPlex formulation F1.

Figure 3 shows photomicrographs of LeciPlex formulation F1. The LeciPlex appears as a discrete, spherical unilamellar vesicle with light internal core and dark bilayer. These results agree with previous reports [26,27,31]. The size obtained by TEM measurements was in the range of 94–250 nm. This size is smaller than that obtained by DLS measurements (Table 2), since DLS measurements give the hydrodynamic diameter of the nanoparticles, which is usually larger than the actual particle size. In contrast, TEM measurements give the size of the vesicles after drying [50].

### 3.5. In Vitro Drug Release Studies 

**Figure 4:**
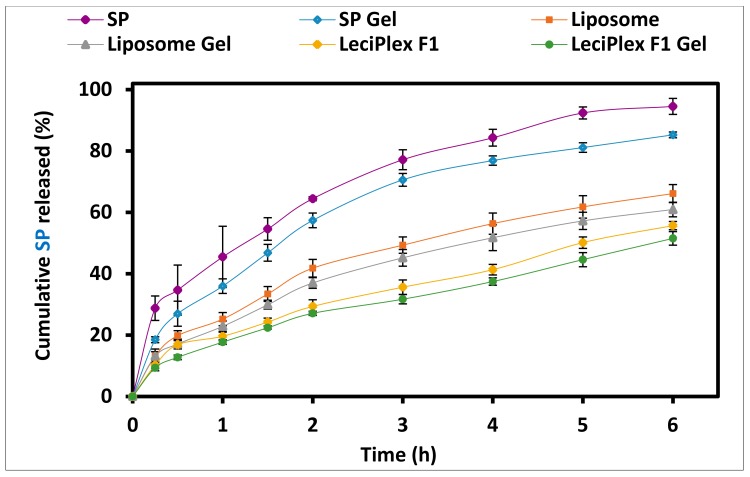
In vitro SP release from different formulations in phosphate-buffered saline pH 7.4.

Figure 4 shows the in vitro SP release from various formulations in phosphate-buffered saline pH 7.4. SP aqueous solubility is 28 µg/mL and all of the release samples contained 1 mg drug [47,51]. To ensure that sink conditions are maintained, a release medium volume of 200 mL was used. SP suspension, used as a control, showed the fastest release among the studied formulations, where almost complete release was observed after 6 h (94.53 ± 2.61%). Cumulative amount of SP released from liposomes after 6 h (66.13 ± 2.93%) was significantly smaller compared with that released from the drug suspension (*p* < 0.05). This might be attributed to drug encapsulation in the liposome bilayer structure, which limits drug diffusion into the external medium [52]. SP LeciPlex formulation F1 showed further significant reduction in the drug release rate compared to liposomes (*p* < 0.05). Thus, after 6 h, the percentage SP released from LeciPlex formulation F1 was 55.70 ± 1.35%. Other studies showed slower drug release rates from LeciPlex formulations compared to drug solutions [31,42]. The reason behind the slower drug release from LeciPlex compared to liposomes is not clear, but might be related to drug interaction with CTAB, which is present in LeciPlex but not in liposomes. This interaction might slow down the drug release rate. Incorporation of any of the tested preparations in 1% MC gel led to a significant reduction in the cumulative amount of drug released after 6 h (*p* < 0.05) compared to the corresponding preparation. For instance, after 6 h, the cumulative percent SP released from SP gel was 85.27 ± 0.95% compared to 94.53 ± 2.61% for SP suspension. Similarly, the cumulative percent released from SP liposome gel and SP liposome were 60.93 ± 2.37% and 66.13 ± 2.93%, respectively. SP LeciPlex F1 gel had the smallest cumulative percent released of 51.53 ± 2.21% after 6 h compared to 55.70 ± 1.35% for LeciPlex F1. The gel network probably creates a diffusion barrier for the drug, which results in lowering the drug release rate. Previous reports showed that vesicular carriers dispersed in gel preparations had slower drug release rates compared with the vesicular dispersions [52,53].

### 3.6. Kinetics of Drug Release

Data of drug release were fitted using various mathematical models, and the model that best describes the data was selected based on the highest correlation coefficient value (*R*^2^) (Table 3). The release rate constant (*K*), which is indicative of the drug release rate, was also calculated for all of the formulations and is listed in Table 3. Release data from SP suspension, SP LeciPlex F1, and SP LeciPlex F1 gel were best fitted by the Hixson–Crowell cube root equation (Table 3). This model is applied when drug release occurs by progressive dissolution, where the size of the particle decreases as the dissolution takes place [38]. The release rate constant (*K*) values were 0.43, 0.16, and 0.14 h^−1^ for SP suspension, SP LeciPlex F1, and SP LeciPlex F1 gel, respectively. This confirms that the release rate followed this order: SP suspension > SP LeciPlex F1 > SP LeciPlex F1 gel, which is consistent with the results shown in Figure 4. Previous studies showed that drug release from nanostructured lipid carriers had high correlation coefficient values for the Hixson–Crowell cube root equation [54]. In contrast, drug release data from SP gel, SP liposomes, and SP liposome gel were best interpreted by the Baker–Lonsdale model (Table 3). This model explains drug release from spherical matrices. Although the Baker–Lonsdale model is based on the Higuchi model, it gave higher *R^2^* values, which emphasizes the importance of nanoparticle shape in controlling the drug release process [55]. The *K* values were 0.04, 0.02 and 0.02 for SP gel, SP liposomes, and SP liposome gel, respectively. This order is in agreement with the drug release profiles shown in Figure 4. Korsmeyer–Peppas equation also gave quite high *R*^2^ values (≥0.9921) and the release exponent (*n*) was in the range of 0.40–0.53. This indicates that the release mechanism from all of the tested formulations is best described by Fickian diffusion, which is dependent on concentration gradient, diffusion distance, and the degree of swelling [56,57]. The *K* values calculated using Korsmeyer–Peppas equation ranged from 0.19 to 0.46 (Table 3) and were consistent with the release rates observed in Figure 4.

### 3.7. Ex Vivo Skin Permeation Studies 

Successful treatment of skin disorders such as acne requires that a substantial amount of the applied drug dose is retained within the skin layers [58,59,60]. However, topically applied drugs might be absorbed in body areas close to the action site, where a local effect is expected. It is therefore, a common practice that both the amount of drug permeating through the skin and that deposited in various layers are measured and reported [27,61,62]. The permeation profiles of SP and skin deposition behavior were tested using excised rat abdominal skin. Ideally this experiment should have been done using human skin, but problems related to availability and storage favored the use of excised animal skin [63]. Rat skin is the most frequently used animal skin model due to its structural similarity to human skin [63]. However, results from these experiments should be carefully interpreted, since previous studies have shown rat skin to be more permeable than human skin [63].

**Figure 5:**
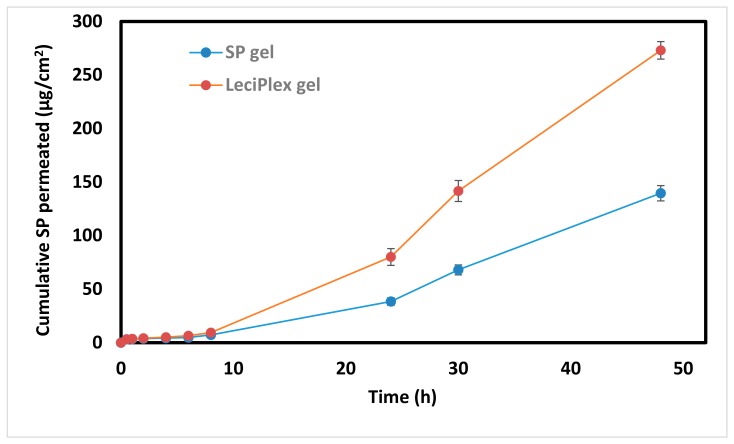
Ex vivo permeation of SP through excised rat abdominal skin from SP gel and SP LeciPlex gel as a function of time.

SP LeciPlex F1 was suspended in methylcellulose gel (1%, *w*/*v*) to facilitate application on the skin and compared with SP gel as a control. Figure 5 shows the cumulative SP amount permeated through the rat skin as a function of time. SP permeability from both formulations was slow up to 8 h, where less than 1% of the applied dose (~0.7 and 0.9% for SP gel and SP LeciPlex gel, respectively) was permeated through the skin. After 8 h, there was a rapid increase in the amount of SP permeated from both formulations. After 24 h, the SP amount permeated from SP LeciPlex gel was around 2-fold higher than that from SP gel (significant difference, *p* < 0.05). At the end of the study (48 h), the cumulative amount permeated from SP LeciPlex gel (273 ± 8.2 µg·cm^−2^) was almost 2-fold higher than that observed for SP gel (139.6 ± 7.2 µg·cm^−2^).

The transdermal flux (taken as the slope of the curve obtained by plotting the cumulative amount of SP permeated through skin vs. time) was calculated for both formulations (Table 4). The flux for SP LeciPlex gel was ~2-fold higher than that of SP gel (3.9 ± 0.1 µg·cm^−2^·h^−1^ vs. 2.0 ± 0.1 µg·cm^−2^·h^−1^, respectively). In addition, the apparent permeability coefficient for SP LeciPlex gel was ~3.5-fold higher than that of SP gel (Table 4). The cumulative SP amounts deposited in the stratum corneum and other skin layers from the LeciPlex gel were about 1.8- and 2.6-fold higher than that from SP control gel, respectively (Table 4). The better SP permeability, flux, and skin deposition observed for the LeciPlex gel might be attributed to several factors. Cationic surface charge of LeciPlex facilitates their interaction with the negatively charged cell surface, which in turn leads to better drug uptake and permeation through the skin [64,65,66]. Further, the small particle size of the LeciPlexes is another important factor that contributes to their enhanced skin permeation. Other LeciPlex formulations have shown enhanced drug permeation through biological membranes [27,31,67].

## 4. Conclusions

SP-loaded LeciPlexes were efficiently prepared by a simple one-step procedure. The LeciPlexes had high drug encapsulation efficiency, positive surface charge, and particle size in the nanometer range. The particles had a spherical, uniform shape and bilayer structure. FT-IR and DSC studies confirmed the incorporation of the drug into the LeciPlexes through hydrophobic and hydrogen-bonding interactions. The LeciPlexes were able to sustain SP release compared with the drug suspension. Incorporation of the LeciPlexes into methylcellulose gel resulted in a further decrease in the drug release rate. The LeciPlexes suspended in methylcellulose gel were able to enhance SP permeability and flux through rat abdominal skin by 2-fold compared with plain SP gel. Further, cumulative SP amounts deposited in the stratum corneum and other skin layers were about 1.8- and 2.6-fold higher than that from SP control gel, respectively. These results confirm the ability of the developed LeciPlex formulation to serve as an efficient topical delivery system for SP with a potential for application in the treatment of female acne. 

## Figures and Tables

**Table 1:** Composition of different SP LeciPlex formulations.

Ingredients	Formula									
	F1	F2	F3	F4	F5	F6	F7	F8	F9	F10
SP (mg)	10	20	30	40	50	10	10	10	10	10
PL-90G (mM)	24	24	24	24	24	24	24	24	24	24
CTAB (mM)	24	24	24	24	24	6	12	36	48	72
Transcutol^®^P (mL)	0.5	0.5	0.5	0.5	0.5	0.5	0.5	0.5	0.5	0.5
Distilled water (mL)	9.5	9.5	9.5	9.5	9.5	9.5	9.5	9.5	9.5	9.5

**Table 2:** LeciPlex particle size, polydispersity index, zeta potential, and drug encapsulation efficiency of different formulations.

Formula	Particle Size (nm)	PDI ^a^	Zeta Potential (mV)	Encapsulation Efficiency (%) ^b^
F1	337.0 ± 11.1	0.3	49.3 ± 3.5	88.7 ± 3.4
F2	668.5 ± 120.3	0.7	57.6 ± 2.1	92.7 ± 5.7
F3	591.0 ± 13.7	0.9	56.9 ± 1.0	93.6 ± 6.9
F4	487.9 ± 14.5	0.5	57.7 ± 3.3	86.6 ± 2.3
F5	439.6 ± 25.1	0.8	54.3 ± 0.2	79.5 ± 7.6
F6	381.5 ± 8.8	0.3	55.2 ± 1.4	85.3 ± 1.9
F7	355.3 ± 9	0.4	51.9 ± 1.2	87.7 ± 5.7
F8	321.5 ± 40.5	0.9	52.0 ± 1.4	92.8 ± 5.7
F9	206.5 ± 100	0.9	54.5 ± 3.7	72.6 ± 7.1
F10	108.0 ± 25.3	0.6	51.6 ± 2.1	65.8 ± 4.8

^a^ PDI, polydispersity index, mean of three different measurements ±SD (standard deviation). ^b^ Percent drug encapsulation efficiency, calculated from Equation (1), mean of three different measurements ±SD.

**Table 3:** Fitting of in vitro release data into different kinetic models.

Formulation	Zero Order		First Order		Higuchi Model		Hixson–Crowell Model		Baker–Lonsdale Model		Korsemeyer–Peppas Equation		
	*R* ^2^	*K*	*R* ^2^	*K*	*R* ^2^	*K*	*R* ^2^	*K*	*R* ^2^	*K*	*R* ^2^	*K*	*n*
SP suspension	0.9662	11.64	−0.9965	0.46	0.9947	36.04	0.9968	0.43	0.9965	0.06	0.9922	0.46	0.40
SP gel	0.9535	11.50	−0.9936	0.30	0.9898	35.91	0.9841	0.33	0.9952	0.04	0.9964	0.38	0.53
SP Liposomes	0.9728	9.05	−0.9935	0.16	0.9967	27.87	0.9881	0.21	0.9987	0.02	0.9963	0.27	0.53
SP Liposome gel	0.9786	8.43	−0.9942	0.14	0.9974	25.83	0.9901	0.18	0.9985	0.02	0.9948	0.25	0.50
SP LeciPlex F1	0.9935	7.50	−0.9968	0.12	0.9932	22.56	0.9968	0.16	0.9848	0.01	0.9921	0.21	0.50
SP LeciPlex F1 gel	0.9937	6.97	−0.9965	0.10	0.9943	20.99	0.9965	0.14	0.9821	0.01	0.9973	0.19	0.53

**Table 4:** Ex vivo permeation parameters of SP from different formulations through rat skin.

Parameter	*Q* ^a^	*Js* ^b^	*P*_app_ × 10^−3 c^	*Q* (SC) ^d^	*Q* (Rest of Skin) ^e^
SP LeciPlex	273.0 ± 8.2	3.9 ± 0.1	14.6 ± 3.7	20.8 ± 2.2	4.1 ± 0.3
Control SP	139.6 ± 7.2	2.0 ± 0.1	4.1 ± 0.4	11.7 ± 1.5	1.6 ± 0.1

^a^ Cumulative amount of SP permeated (µg·cm^−2^) after 48 h. ^b^ Flux (permeation rate constant) at steady state (µg·cm^−2^·h^−1^), obtained from the slope of the regression line obtained from plotting amount of SP permeated vs. time. ^c^ Apparent permeability coefficient (cm·h^−1^) calculated from Equation (2). ^d^ Amount deposited in stratum corneum after 48 h. ^e^ Amount deposited in rest of skin after 48 h.
